# Optimal Filtering Methods to Structural Damage Estimation under Ground Excitation

**DOI:** 10.1155/2013/528109

**Published:** 2013-12-17

**Authors:** Chien-Shu Hsieh, Der-Cherng Liaw, Tzu-Hsuan Lin

**Affiliations:** ^1^Department of Electrical and Electronic Engineering, Ta Hwa University of Science and Technology, Hsinchu 30740, Taiwan; ^2^Department of Electrical Engineering, National Chiao Tung University, Hsinchu 30010, Taiwan; ^3^Sinotech Engineering Consultants, Inc., Taipei 10570, Taiwan

## Abstract

This paper considers the problem of shear building damage estimation subject to earthquake ground excitation using the Kalman filtering approach. The structural damage is assumed to take the form of reduced elemental stiffness. Two damage estimation algorithms are proposed: one is the multiple model approach via the optimal two-stage Kalman estimator (OTSKE), and the other is the robust two-stage Kalman filter (RTSKF), an unbiased minimum-variance filtering approach to determine the locations and extents of the damage stiffness. A numerical example of a six-storey shear plane frame structure subject to base excitation is used to illustrate the usefulness of the proposed results.

## 1. Introduction

Structural health monitoring (SHM) is a rapidly developing field encompassing technology and algorithms for sensing the state of a structural system, diagnosing the structure's current condition, performing a prognosis of expected future performance, and providing information for decisions about maintenance, safety, and emergency actions [1, 2]. Advances in microelectromechanical system (MEMS) technology over the past decade have provided opportunities for sensing, wireless communication, and distributed data processing for a variety of new SHM applications [2].

A building structure may sustain damage during a strong earthquake. Monitoring the structural health of buildings has thus received considerable interest over the last decade. The vibration-based damage detection technique is one of the more promising fields in SHM because it is nondestructive and the vibration signal of a structure is easily measurable using properly deployed sensors [3, 4]. Structural damage may be detected through the variation of structural features, such as natural frequencies, modal damping, mode shapes, frequency response functions (FRFs), and stiffness and flexibility matrices [3, 5]. In this study, it is assumed that only the stiffness matrix is altered when the structural system is damaged.

An FRF expresses the structural response to an applied force as a function of frequency. Theoretically, FRFs can be expressed in terms of system properties of mass, stiffness, damping, and modal properties. It is known that damage detection algorithms using FRFs exhibit several advantages, and have been applied to damage detection by many researchers (see [6] and the references therein). However, as noted by [7, 8], detection using FRFs may suffer from some disadvantages due to the fact that it is a frequency domain approach. Moreover, it is an indirect approach to detecting the damage location and extent due to the need to transform the sensor data into measured FRFs. Thus, suitable chosen FRFs and frequencies that are close to the natural frequencies of the damaged system are required in order to solve the damage detection problem [3, 9]. On the other hand, this research focuses on a direct approach to detecting structural damage using the Kalman filtering method, which is a time-domain approach.

Unknown input filtering (UIF) has played a significant role in many applications, for example, bias compensation [10, 11], maneuvering target tracking [12, 13], geophysical and environmental applications [14], fault detection and isolation problems [15], and functional filtering [16]. Note that for the first two of the above applications some assumptions of the unknown input are used, while the remaining applications are solved by assuming that no prior information about the unknown input is available.

In this study, based on the equation of motion under ground excitation, a structural damage detection and identification problem is formulated as a standard state-space system with unknown inputs, where the unknown input vector represents the extent of the damage. Treating the unknown input vector as a stochastic process with a given wide-sense representation, and augmenting it with the system state, an augmented state Kalman filter (ASKF) or, equivalently, the optimal two-stage Kalman estimator (OTSKE) [11] may be used to produce the optimal unknown input estimate, and thus the extent of the damage can be identified. Damage locations can, therefore, also be detected. However, it is well known that the optimality of the ASKF (or OTSKE) can be compromised by a poor choice of unknown input model. Thus, the damage detection may yield a false-alarm effect. On the other hand, without assuming the unknown input model, the unbiased minimum variance filtering (UMVF) in [17–19] serves as an effective method for yielding the optimal unknown input and state estimates. Note that the result in [18] is applicable only to a case in which the direct feedthrough matrix of the unknown input to the output has full-column rank, the limitation of which, however, can be relaxed by using the refined version given in [20]. Furthermore, the descriptor Kalman filtering (DKF) [21] also serves as a useful means to estimate the system state for systems with unknown inputs [22].

This paper aims to present some damage estimation methods based on the Kalman filtering approach to determine the location and extent of structural damage under ground excitation. Specifically, the objective of this paper is to design damage detection and identification algorithms for detecting the location and extent of structural damage. In the following section, two damage estimation algorithms are proposed: one is the multiple model approach via the OTSKE, and the other is a specific discrete-time Kalman filtering algorithm, called the robust two-stage Kalman filter (RTSKF) [17], to determine the location and extent of damage stiffness. The usefulness of the proposed results is verified using a numerical example of a six-storey shear plane frame structure subject to base excitation.

The remainder of this paper is organized as follows.  Section 2 states the estimation problem considered in this paper.  Section 3 formulates a discrete-time state space model for the considered problem in order to facilitate the estimator design.  Section 4 derives two optimal unknown input estimators, called the ASKF and the OTSKE, which can simultaneously estimate the state and the extent of structural damage subject to the latter being effectively described as a random-walk process.  Section 5 presents two unbiased minimum-variance unknown input estimators for optimal damage stiffness estimation without resorting to the assumption of a specific unknown input model. The application of the proposed optimal estimators to the damage location detection and damage stiffness identification in a structurally damaged system is explored in Section 6. A numerical example demonstrating the usefulness of the proposed results is given in Section 7.  Section 8 concludes the paper. This paper is an extended and refined version of conference papers [23, 24].

## 2. Problem Formulation

Consider a one-dimensional shear building with *n* degree of freedom under ground excitation as follows:

(1) $$\begin{array}{c} M\ddot{x}+C\dot{x}+Kx=-ML{\ddot{x}}_{g}, \end{array}$$

where *x* ∈ *R* ^*n*^ is the displacement response vector and ${\ddot{x}}_{g}\in R$ is the ground acceleration excitation; *L* represents the loading vector, given by *L* = [1 1 ⋯ 1]^*T*^ and *M*, *C*, and *K* represent the *n* × *n* mass, damping, and stiffness matrices, which are given, respectively, as follows:

(2) $$\begin{array}{c} M=\left[\begin{array}{ccccc} m_{1} & & & & \\ & m_{2} & & & \\ & & \ddots & & \\ & & & m_{n-1} & \\ & & & & m_{n} \end{array}\right],\\ C=\left[\begin{array}{ccccc} c_{1}+c_{2} & -c_{2} & & & \\ -c_{2} & c_{2}+c_{3} & & & \\ & & \ddots & & \\ & & & c_{n-1}+c_{n} & -c_{n}\\ & & & -c_{n} & c_{n} \end{array}\right],\\ K=\left[\begin{array}{ccccc} k_{1}+k_{2} & -k_{2} & & & \\ -k_{2} & k_{2}+k_{3} & & & \\ & & \ddots & & \\ & & & k_{n-1}+k_{n} & -k_{n}\\ & & & -k_{n} & k_{n} \end{array}\right]. \end{array}$$

It is assumed in this paper that the variation of mass and damping matrices are unchanged after the system is damaged. Therefore, the stiffness matrix for the damaged system is represented as *K*_*d*_ = *K* + Δ*K*, where Δ*K* is obtained from *K* using the damage substitution *k*_*i*_ → *k*_*i*_ *δk*_*i*_. Define the unknown input vector *d* as

(3) $$\begin{array}{c} d={\left[\begin{array}{cccc} \delta k_{1} & \delta k_{2} & \cdots & \delta k_{n} \end{array}\right]}^{T}. \end{array}$$

Then, the dynamics of system (1) corresponding to the above damaged system can be obtained as follows:

(4) $$\begin{array}{c} M\ddot{x}+C\dot{x}+Kx=-ML{\ddot{x}}_{g}-F\left(x\right)d, \end{array}$$

where

(5) F(x)=[1−11−1⋱⋱1−11]×diag⁡{k1x1,k2(x2−x1),…,kn(xn−xn−1)}.

In realizing the well known usefulness of digital technology, the sensor output is obtained by the following discrete-time measurement equation:

(6) $$\begin{array}{c} y_{k}=H_{d}x\left(kT\right)+H_{v}\dot{x}\left(kT\right)+H_{a}\ddot{x}\left(kT\right), \end{array}$$

where *T* is the sampling period of the sensor. The problem of interest in this paper is then to design an optimal discrete-time unknown input estimator ${\hat{d}}_{k}$ for the damage of the stiffness matrix based on the Kalman filtering approach. To achieve this goal, a discrete-time state-space model corresponding to system (4) and measurement (6) is first formed, which is detailed in the following section.

## 3. Discrete-Time State-Space Model

Defining the augmented state vector *X* as ${\left[x^{T}\;{\dot{x}}^{T}\right]}^{T}$, the damaged system (4) can be rewritten as follows:

(7) $$\begin{array}{c} \dot{X}=AX+Bu+G\left(x\right)d, \end{array}$$

where $u={\ddot{x}}_{g}$,

(8) $$\begin{array}{c} A=\left[\begin{array}{cc} 0 & I\\ -M^{-1}K & -M^{-1}C \end{array}\right],\\ B=\left[\begin{array}{c} 0\\ -L \end{array}\right],\;\;G\left(x\right)=\left[\begin{array}{c} 0\\ -M^{-1}F\left(x\right) \end{array}\right]. \end{array}$$

Next, the known input *u* is assumed to be a piecewise constant during the sampling interval. Thus, sampling the continuous-time system (7) and using the discrete-time measurement (6) gives the following approximated discrete-time state-space model:

(9) $$\begin{array}{c} X_{k+1}=A_{s}X_{k}+B_{s}u_{k}+G_{k}d_{k}, \end{array}$$

(10) $$\begin{array}{c} y_{k}=\Phi X_{k}+Du_{k}+H_{k}d_{k}, \end{array}$$

where

(11) $$\begin{array}{c} X_{k}=X\left(kT\right),\;\;A_{s}=I+AT,\;\;B_{s}=BT,\\ u_{k}=u\left(kT\right),\;\;G_{k}=G\left(x\left(kT\right)\right)T,\;\;d_{k}=d\left(kT\right),\\ \Phi =\left[\begin{array}{cc} H_{d}-H_{a}M^{-1}K & H_{v}-H_{a}M^{-1}C \end{array}\right],\\ D=-H_{a}L,\;\;H_{k}=-H_{a}M^{-1}F\left(x\left(kT\right)\right). \end{array}$$

Because the displacement vector *x* may not be accessible, the matrix *F*(*x*) is therefore unknown, and thus neither matrices *G*_*k*_ or *H*_*k*_ are obtainable. To remedy this problem, the matrices *G*_*k*_ and *H*_*k*_ are implemented alternatively as follows:

(12) $$\begin{array}{c} {\hat{G}}_{k}=\left[\begin{array}{c} 0\\ -M^{-1}F\left({\hat{x}}_{k}\right) \end{array}\right]T,\\ {\hat{H}}_{k}=-H_{a}M^{-1}F\left({\hat{x}}_{k-1}\right), \end{array}$$

where ${\hat{x}}_{k}=\hat{x}(kT)$ is the estimator of the displacement *x*(*kT*), which remains to be determined. Note that ${\hat{H}}_{k}$ is implemented using a one step delay of the state estimation because ${\hat{x}}_{k}$ is dependent on the value of ${\hat{H}}_{k}$. Using (12), the discrete-time systems (9) and (10) can be rewritten as follows:

(13) $$\begin{array}{c} X_{k+1}=A_{s}X_{k}+B_{s}u_{k}+{\hat{G}}_{k}d_{k}+w_{k},\\ y_{k}=\Phi X_{k}+Du_{k}+{\hat{H}}_{k}d_{k}+v_{k}, \end{array}$$

where

(14) $$\begin{array}{c} w_{k}=\left(G_{k}-{\hat{G}}_{k}\right)d_{k},\;\;v_{k}=\left(H_{k}-{\hat{H}}_{k}\right)d_{k}. \end{array}$$

In this paper, *w*_*k*_ and *v*_*k*_ are viewed as independent processes and measurement white noises with covariances *Q*_*k*_^*X*^ and *R*_*k*_, respectively.

## 4. Optimal Unknown Input Estimators Design

In this section, we demonstrate the application of the conventional Kalman filtering approach to identify the damage stiffness vector *d*_*k*_ based on the system (13). To facilitate the following discussions, we assume that the unknown input vector *d*_*k*_ can be described by the following random-walk process:

(15) $$\begin{array}{c} d_{k+1}=d_{k}+w_{k}^{d}, \end{array}$$

where *w*_*k*_^*d*^ is a zero-mean white noise sequence with the following covariances: *E*{*w*_*k*_^*d*^(*w*_*l*_^*d*^)^*T*^} = *Q*_*k*_^*d*^ *δ*_*kl*_, *E*{*w*_*k*_^*d*^ *w*_*l*_^*T*^} = 0, and *E*{*w*_*k*_^*d*^ *v*_*l*_^*T*^} = 0.

### 4.1. Design via the ASKF

In this subsection, we show the result of applying the well-known ASKF [11] to the damaged system (13)–(15) to identify the damage stiffness vector.

Usings (15), the system of (13) can be augmented as follows:

(16) $$\begin{array}{c} X_{k+1}^{a}={\overset{-}{A}}_{k}X_{k}^{a}+\overset{-}{B}u_{k}+W_{k},\\ y_{k}={\overset{-}{H}}_{k}X_{k}^{a}+Du_{k}+v_{k}, \end{array}$$

where

(17) $$\begin{array}{c} X_{\left(\cdot \right)}^{a}=\left[\begin{array}{c} X_{\left(\cdot \right)}\\ d_{\left(\cdot \right)} \end{array}\right],\;\;{\overset{-}{A}}_{k}=\left[\begin{array}{cc} A_{s} & {\hat{G}}_{k}\\ 0 & I \end{array}\right], \end{array}$$

(18) $$\begin{array}{c} \overset{-}{B}=\left[\begin{array}{c} B_{s}\\ 0 \end{array}\right],\;\;W_{k}=\left[\begin{array}{c} w_{k}\\ w_{k}^{d} \end{array}\right],\;\;{\overset{-}{H}}_{k}=\left[\begin{array}{cc} \Phi & {\hat{H}}_{k} \end{array}\right]. \end{array}$$

Solving (16) for *X*_*k*∣*k*_^*a*^ using the ASKF, we obtain

(19) $$\begin{array}{c} X_{k\;|\;k-1}^{a}={\overset{-}{A}}_{k-1}X_{k-1\;|\;k-1}^{a}+\overset{-}{B}u_{k-1},\\ X_{k\;|\;k}^{a}=X_{k\;|\;k-1}^{a}+K_{k}\left(y_{k}-Du_{k}-{\overset{-}{H}}_{k}X_{k\;|\;k-1}^{a}\right),\\ P_{k\;|\;k-1}={\overset{-}{A}}_{k-1}P_{k-1\;|\;k-1}{\overset{-}{A}}_{k-1}^{T}+Q_{k-1},\\ K_{k}=P_{k\;|\;k-1}{\overset{-}{H}}_{k}^{T}{\left({\overset{-}{H}}_{k}P_{k\;|\;k-1}{\overset{-}{H}}_{k}^{T}+R_{k}\right)}^{-1},\\ P_{k\;|\;k}=\left(I-K_{k}{\overset{-}{H}}_{k}\right)P_{k\;|\;k-1}, \end{array}$$

where

(20) $$\begin{array}{c} P_{\left(\cdot \right)}=\left[\begin{array}{cc} P_{\left(\cdot \right)}^{X} & P_{\left(\cdot \right)}^{Xd}\\ P_{\left(\cdot \right)}^{dX} & P_{\left(\cdot \right)}^{d} \end{array}\right],\;\;Q_{k}=\left[\begin{array}{cc} Q_{k}^{X} & 0\\ 0 & Q_{k}^{d} \end{array}\right]. \end{array}$$

Finally, the damage stiffness vector *d*_*k*_ and the displacement *x*_*k*_ can be estimated, respectively, from the above ASKF as

(21) $$\begin{array}{c} {\hat{d}}_{k}=\left[\begin{array}{ccc} 0 & 0 & I \end{array}\right]X_{k\;|\;k}^{a},\\ {\hat{x}}_{k}=\left[\begin{array}{ccc} I & 0 & 0 \end{array}\right]X_{k\;|\;k}^{a}. \end{array}$$

### 4.2. Design via the OTSKE

It is noted that the computational complexity of the ASKF can be reduced using the previously proposed OTSKE (see [11] for details). In the following, we show the result of applying the OTSKE to the damaged systems (13)–(15) to identify the damage stiffness vector.

Using the following two-stage *U*-*V* transformation:

(22) $$\begin{array}{c} X_{k\;|\;k-1}^{a}=T\left(U_{k}\right){\overset{-}{X}}_{k\;|\;k-1}^{a},\\ X_{k\;|\;k}^{a}=T\left(V_{k}\right){\overset{-}{X}}_{k\;|\;k}^{a},\\ P_{k\;|\;k-1}=T\left(U_{k}\right){\overset{-}{P}}_{k\;|\;k-1}{\left(T\left(U_{k}\right)\right)}^{T},\\ K_{k}=T\left(V_{k}\right){\overset{-}{K}}_{k},\\ P_{k\;|\;k}=T\left(V_{k}\right){\overset{-}{P}}_{k\;|\;k}{\left(T\left(V_{k}\right)\right)}^{T}, \end{array}$$

where

(23) $$\begin{array}{c} {\overset{-}{X}}_{\left(\cdot \right)}^{a}=\left[\begin{array}{c} {\overset{-}{X}}_{\left(\cdot \right)}\\ d_{\left(\cdot \right)} \end{array}\right],\;\;{\overset{-}{P}}_{\left(\cdot \right)}=\left[\begin{array}{cc} P_{\left(\cdot \right)}^{\overset{-}{X}} & 0\\ 0 & P_{\left(\cdot \right)}^{d} \end{array}\right],\\ {\overset{-}{K}}_{k}=\left[\begin{array}{c} K_{k}^{\overset{-}{X}}\\ K_{k}^{d} \end{array}\right],\;\;T\left(M\right)=\left[\begin{array}{cc} I & M\\ 0 & I \end{array}\right], \end{array}$$

from (19), we can obtain the OTSKE as follows:

(24) $$\begin{array}{c} X_{k\;|\;k}={\overset{-}{X}}_{k\;|\;k}+V_{k}d_{k\;|\;k}, \end{array}$$

where ${\overset{-}{X}}_{k|k}$ is given by

(25) X−k ∣ k−1=AsX−k−1 ∣ k−1+Bsuk−1 +(U−k−Uk)dk−1 ∣ k−1,X−k ∣ k=X−k ∣ k−1+KkX−(yk−Duk−ΦX−k ∣ k−1),Pk ∣ k−1X−=AsPk−1 ∣ k−1X−AsT+Qk−1X+UkQk−1dU−kT,KkX−=Pk ∣ k−1X−ΦTR−k−1,R−k=ΦPk ∣ k−1X−ΦT+Rk,Pk ∣ kX−=(I−KkX−Φ)Pk ∣ k−1X−,

*d*_*k*∣*k*_ is given by

(26) dk ∣ k=(I−KkdSk)dk−1 ∣ k−1 +Kkd(yk−Duk−ΦX−k ∣ k−1),Pk ∣ k−1d=Pk−1 ∣ k−1d+Qk−1d,Kkd=Pk ∣ k−1dSkT(SkPk ∣ k−1dSkT+R−k)−1,Pk ∣ kd=(I−KkdSk)Pk ∣ k−1d,

and the blending matrices ${\overset{-}{U}}_{k}$, *U*_*k*_, *V*_*k*_, and *S*_*k*_ are given, respectively, by

(27) $$\begin{array}{c} {\overset{-}{U}}_{k}=A_{s}V_{k-1}+{\hat{G}}_{k-1},\\ U_{k}={\overset{-}{U}}_{k}P_{k-1\;|\;k-1}^{d}{\left(P_{k\;|\;k-1}^{d}\right)}^{-1},\\ V_{k}=U_{k}-K_{k}^{\overset{-}{X}}S_{k},\\ S_{k}=\Phi U_{k}+{\hat{H}}_{k}. \end{array}$$

Finally, the damage stiffness vector *d*_*k*_ and the displacement *x*_*k*_ can be estimated from the above OTSKE as follows:

(28) $$\begin{array}{c} {\hat{d}}_{k}=d_{k\;|\;k},\;\;{\hat{x}}_{k}=\left[\begin{array}{cc} I & 0 \end{array}\right]X_{k\;|\;k}. \end{array}$$

## 5. Unbiased Minimum-Variance Unknown Input Estimators Design

In this section, we show how to derive an optimal damage stiffness estimator without resorting to the assumption of a specific unknown input model, for example, (15), usually required in the standard Kalman filtering approach. To achieve this goal, we consider the previously proposed extended DKF (EDKF) [22], which can be viewed as a robust version of the ASKF applied to the system of (13). Furthermore, we show that the state estimator of the EDKF can be implemented alternatively in the form of the RTSKF developed in [17], which can be viewed as a robust version of the OTSKE.

### 5.1. Design via the EDKF

First, we reformulate system (13) as the following descriptor system (see [22] for details):

(29) $$\begin{array}{c} EX_{k+1}^{a}={\hat{A}}_{k}X_{k}^{a}+B_{s}u_{k}+{\hat{U}}_{k}d_{k}+w_{k}, \end{array}$$

(30) $$\begin{array}{c} y_{k}={\overset{-}{H}}_{k}X_{k}^{a}+Du_{k}+v_{k}, \end{array}$$

where

(31) $$\begin{array}{c} E=\left[\begin{array}{cc} I & 0 \end{array}\right],\;\;{\hat{A}}_{k}=\left[\begin{array}{cc} A_{s} & {\hat{G}}_{k} \end{array}\right],\\ {\hat{U}}_{k}={\hat{G}}_{k}\left(I-{\hat{H}}_{k}^{+}{\hat{H}}_{k}\right), \end{array}$$

and *X*_*k*_^*a*^ and ${\overset{-}{H}}_{k}$ are defined in (17)-(18). Here, the notation *M* ^+^ is the Moore-Penrose pseudoinverse of *M*.

Next, using the following full-rank factorization:

(32) $$\begin{array}{c} {\hat{U}}_{k}={\overset{-}{\hat{U}}}_{k}{\tilde{\hat{U}}}_{k}, \end{array}$$

where ${\overset{-}{\hat{U}}}_{k}$ is of full-column rank; the descriptor systems (29) and (30) can be rewritten as the following augmented output equation (AOE):

(33) $$\begin{array}{c} \left[\begin{array}{c} {\overset{-}{X}}_{k}^{a}\\ y_{k}-Du_{k} \end{array}\right]=\left[\begin{array}{cc} E & -{\overset{-}{\hat{U}}}_{k-1}\\ {\overset{-}{H}}_{k} & 0 \end{array}\right]\left[\begin{array}{c} X_{k}^{a}\\ {\tilde{d}}_{k-1} \end{array}\right]+\left[\begin{array}{c} \eta_{k-1}\\ v_{k} \end{array}\right], \end{array}$$

where

(34) $$\begin{array}{c} {\overset{-}{X}}_{k}^{a}={\hat{A}}_{k-1}X_{k-1\;|\;k-1}^{a}+B_{s}u_{k-1},\\ \eta_{k}=-{\hat{A}}_{k}\left(X_{k}^{a}-X_{k\;|\;k}^{a}\right)-w_{k},\\ {\tilde{d}}_{k}={\tilde{\hat{U}}}_{k}d_{k}. \end{array}$$

Note that in the above equation, ${\tilde{d}}_{k}$ represents the part of *d*_*k*_ that is unestimable at time *k*.

Then, solving (33) for the estimates of *X*_*k*_^*a*^ and ${\tilde{d}}_{k-1}$, we obtain

(35) [Xk ∣ kad~k−1 ∣ k]=[00I0000I]Λk+×[(X−ka)T(yk−Duk)T00]T,Pk ∣ kXa=−[00I0]Λk+[00I0]T,Pk−1 ∣ kd~=−[000I]Λk+[000I]T,

where

(36) $$\begin{array}{c} \Lambda_{k}=\left[\begin{array}{cccc} {\overset{-}{P}}_{k}^{X^{a}} & 0 & E & -{\overset{-}{\hat{U}}}_{k-1}\\ 0 & R_{k} & {\overset{-}{H}}_{k} & 0\\ E^{T} & {\overset{-}{H}}_{k}^{T} & 0 & 0\\ -{\overset{-}{\hat{U}}}_{k-1}^{T} & 0 & 0 & 0 \end{array}\right], \end{array}$$

with

(37) $$\begin{array}{c} {\overset{-}{P}}_{k}^{X^{a}}={\hat{A}}_{k-1}P_{k-1\;|\;k-1}^{X^{a}}{\hat{A}}_{k-1}^{T}+Q_{k-1}^{X}. \end{array}$$

Finally, the displacement estimate ${\hat{x}}_{k}$ is obtained as follows:

(38) $$\begin{array}{c} {\hat{x}}_{k}=\left[\begin{array}{ccc} I & 0 & 0 \end{array}\right]X_{k\;|\;k}^{a}, \end{array}$$

the estimable unknown input functional estimate *d*_*k*∣*k*_ is given by

(39) $$\begin{array}{c} d_{k\;|\;k}=\left[\begin{array}{ccc} 0 & 0 & I \end{array}\right]X_{k\;|\;k}^{a}, \end{array}$$

and the unestimable unknown input functional estimate ${\tilde{d}}_{k-1|k}$ is given by

(40) $$\begin{array}{c} {\tilde{d}}_{k-1\;|\;k}={\tilde{\hat{U}}}_{k-1}d_{k-1\;|\;k}. \end{array}$$

Using the fact that *d*_*k*∣*k*_ = *φ*_*k*_ *d*_*k*∣*k*_ (see [20] for details), where $\varphi_{k}={\hat{H}}_{k}^{+}{\hat{H}}_{k}$, and subject to the following condition:

(41) $$\begin{array}{c} {\tilde{d}}_{k}\approx {\tilde{d}}_{k-1}, \end{array}$$

which indicates that the unestimable unknown inputs are varied smoothly, the damage stiffness estimate at time *k* can be obtained as follows:

(42) $$\begin{array}{c} {\hat{d}}_{k}=\psi_{k}^{+}\left[\begin{array}{c} d_{k\;|\;k}\\ {\tilde{d}}_{k-1\;|\;k} \end{array}\right],\;\;\psi_{k}=\left[\begin{array}{c} \varphi_{k}\\ {\tilde{\hat{U}}}_{k-1} \end{array}\right]. \end{array}$$

**Remark 1**

The above EDKF can be viewed as a robust version of the ASKF in Section 4.1. Specifically, if the following substitutions are used:

(43) $$\begin{array}{c} E⟵I,\;\;{\hat{A}}_{k}⟵{\overset{-}{A}}_{k},\\ Q_{k}^{X}⟵Q_{k},\;\;B_{s}⟵\overset{-}{B}, \end{array}$$

then the EDKF becomes the ASKF.

**Remark 2**

If the matrix ${\hat{H}}_{k}$ is of full-column rank, then the unknown input vector *d*_*k*_ is completely estimable at time *k*, and one therefore has *φ*_*k*_ = *I* and ${\tilde{\hat{U}}}_{k-1}=0$. Thus, from (42) we have ${\hat{d}}_{k}=[0\;0\;I]X_{k|k}^{a}$.

### 5.2. Design via the RTSKF

Although the EDKF is simple, as is the ASKF, it may also suffer from computational complexity due to the heavy burden of the pseudoinverse operation. In this section, we show how to derive a compact version of the EDKF, which is in the form of the RTSKF.

First, using the approach in [25], we can reform the descriptor system (29) as follows:

(44) Xk+1a=E+(A^kXka+Bsuk+U^−kd~k+wk)+UkXadkXa,

where

(45) $$\begin{array}{c} U_{k}^{X^{a}}={\left[\begin{array}{cc} 0 & I \end{array}\right]}^{T},\;\;d_{k}^{X^{a}}=d_{k+1}. \end{array}$$

Using the following notations:

(46) $$\begin{array}{c} {\overset{˘}{U}}_{k}=\left[\begin{array}{cc} 0 & {\overset{-}{\hat{U}}}_{k}\\ I & 0 \end{array}\right],\;\;{\overset{˘}{d}}_{k}=\left[\begin{array}{c} d_{k+1}\\ {\tilde{d}}_{k} \end{array}\right], \end{array}$$

(44) can be rewritten as

(47) $$\begin{array}{c} X_{k+1}^{a}=E^{+}\left({\hat{A}}_{k}X_{k}^{a}+B_{s}u_{k}+w_{k}\right)+{\overset{˘}{U}}_{k}{\overset{˘}{d}}_{k}. \end{array}$$

Second, using the relationship *E* ^+^ = *E* ^*T*^ and applying the RTSKF [17] to (30) and (47), we have

(48) $$\begin{array}{c} X_{k\;|\;k}^{a}=\left[\begin{array}{c} {\overset{-}{X}}_{k\;|\;k}\\ 0 \end{array}\right]+{\overset{˘}{V}}_{k}{\overset{˘}{d}}_{k-1\;|\;k},\\ P_{k\;|\;k}^{X^{a}}=\left[\begin{array}{cc} P_{k\;|\;k}^{\overset{-}{X}} & 0\\ 0 & 0 \end{array}\right]+{\overset{˘}{V}}_{k}P_{k-1\;|\;k}^{\overset{˘}{d}}{\overset{˘}{V}}_{k}^{T}, \end{array}$$

where ${\overset{-}{X}}_{k|k}$ is given by

(49) $$\begin{array}{c} {\overset{-}{X}}_{k\;|\;k-1}=A_{s}X_{k-1\;|\;k-1}+B_{s}u_{k-1}+{\hat{G}}_{k-1}d_{k-1\;|\;k-1},\\ {\overset{-}{X}}_{k\;|\;k}={\overset{-}{X}}_{k\;|\;k-1}+K_{k}^{\overset{-}{X}}\left(y_{k}-Du_{k}-\Phi {\overset{-}{X}}_{k\;|\;k-1}\right),\\ P_{k\;|\;k-1}^{\overset{-}{X}}={\hat{A}}_{k-1}P_{k-1\;|\;k-1}^{X^{a}}{\hat{A}}_{k-1}^{T}+Q_{k-1}^{X},\\ K_{k}^{\overset{-}{X}}=P_{k\;|\;k-1}^{\overset{-}{X}}\Phi^{T}{\tilde{R}}_{k}^{-1},\\ P_{k\;|\;k}^{\overset{-}{X}}=\left(I-K_{k}^{\overset{-}{X}}\Phi \right)P_{k\;|\;k-1}^{\overset{-}{X}}, \end{array}$$

${\overset{˘}{d}}_{k-1|k}$ is given by

(50) $$\begin{array}{c} {\overset{˘}{d}}_{k-1\;|\;k}=K_{k}^{\overset{˘}{d}}\left(y_{k}-Du_{k}-\Phi {\overset{-}{X}}_{k\;|\;k-1}\right),\\ K_{k}^{\overset{˘}{d}}=P_{k-1\;|\;k}^{\overset{˘}{d}}S_{k}^{T}{\tilde{R}}_{k}^{-1},\\ P_{k-1\;|\;k}^{\overset{˘}{d}}={\left(S_{k}^{T}{\tilde{R}}_{k}^{-1}S_{k}\right)}^{+}, \end{array}$$

and ${\overset{˘}{V}}_{k}$, *S*_*k*_, and ${\tilde{R}}_{k}$ are given, respectively, by

(51) $$\begin{array}{c} {\overset{˘}{V}}_{k}=\left[\begin{array}{c} V_{k}\\ \left[\begin{array}{cc} I & 0 \end{array}\right] \end{array}\right],\;\;V_{k}=\left[\begin{array}{cc} 0 & {\overset{-}{\hat{U}}}_{k-1} \end{array}\right]-K_{k}^{\overset{-}{X}}S_{k}, \end{array}$$

(52) $$\begin{array}{c} S_{k}=\left[\begin{array}{cc} {\hat{H}}_{k} & \Phi {\overset{-}{\hat{U}}}_{k-1} \end{array}\right], \end{array}$$

(53) $$\begin{array}{c} {\tilde{R}}_{k}=\Phi P_{k\;|\;k-1}^{\overset{-}{X}}\Phi^{T}+R_{k}. \end{array}$$

Third, using (17), (46), (48), and (51), we have

(54) $$\begin{array}{c} X_{k\;|\;k}={\overset{-}{X}}_{k\;|\;k}+V_{k}{\overset{˘}{d}}_{k-1\;|\;k}, \end{array}$$

(55) $$\begin{array}{c} d_{k\;|\;k}=\left[\begin{array}{cc} I & 0 \end{array}\right]{\overset{˘}{d}}_{k-1\;|\;k}, \end{array}$$

(56) $$\begin{array}{c} {\tilde{d}}_{k-1\;|\;k}=\left[\begin{array}{cc} 0 & I \end{array}\right]{\overset{˘}{d}}_{k-1\;|\;k}, \end{array}$$

which have the following respective error covariances:

(57) $$\begin{array}{c} P_{k\;|\;k}^{X}=P_{k\;|\;k}^{\overset{-}{X}}+V_{k}P_{k-1\;|\;k}^{\overset{˘}{d}}V_{k}^{T},\\ P_{k\;|\;k}^{d}=\left[\begin{array}{cc} I & 0 \end{array}\right]P_{k-1\;|\;k}^{\overset{˘}{d}}{\left[\begin{array}{cc} I & 0 \end{array}\right]}^{T},\\ P_{k-1\;|\;k}^{\tilde{d}}=\left[\begin{array}{cc} 0 & I \end{array}\right]P_{k-1\;|\;k}^{\overset{˘}{d}}{\left[\begin{array}{cc} 0 & I \end{array}\right]}^{T}. \end{array}$$

Finally, the displacement estimate ${\hat{x}}_{k}$ is obtained as follows:

(58) $$\begin{array}{c} {\hat{x}}_{k}=\left[\begin{array}{cc} I & 0 \end{array}\right]X_{k\;|\;k}, \end{array}$$

and the damage stiffness estimate at time *k* is obtained as follows:

(59) $$\begin{array}{c} {\hat{d}}_{k}=\psi_{k}^{+}{\overset{˘}{d}}_{k-1\;|\;k}. \end{array}$$

The equivalence of the EDKF and the RTSKF can be verified as shown in [25].

**Remark 3**

The above RTSKF can be viewed as a robust version of the OTSKE in Section 4.2 and is an extended result of the original in [17]. Specifically, if the unknown inputs do not enter the measurement equation, that is, ${\hat{H}}_{k}=0$, one has *φ*_*k*_ = 0, $S_{k}=\Phi {\overset{-}{\hat{U}}}_{k-1}$, ${\overset{-}{\hat{U}}}_{k}={\hat{G}}_{k}$, and ${\tilde{\hat{U}}}_{k}=I$. Thus, from (50), (55), and (56), we have *d*_*k*∣*k*_ = 0 and ${\overset{˘}{d}}_{k-1|k}=d_{k-1|k}$, which signifies that only one delay of the unestimable unknown input estimate can be obtained. Note that in this extended RTSKF, the filter ${\overset{˘}{d}}_{k-1|k}$ serves as a primitive unknown input estimator in the sense that through it both the estimable and unestimable unknown input estimates, that is, *d*_*k*∣*k*_ and ${\tilde{d}}_{k-1|k}$, respectively, can be obtained.

**Remark 4**

If the matrix ${\hat{H}}_{k}$ is of full-column rank, then one has ${\overset{-}{\hat{U}}}_{k}=0$, ${\tilde{\hat{U}}}_{k}=0$, and $S_{k}={\hat{H}}_{k}$. Then, from (55) and (59), we have ${\hat{d}}_{k}=d_{k|k}$, and hence the obtained RTSKF will be equivalent to the RTSF originally developed in [18]. Thus, the above RTSKF can be viewed as an extended result of those in [18, 20].

## 6. Damage Detection and Identification

In this section, we demonstrate the application of the previously proposed optimal unknown input estimators to simultaneously detect and identify the damage stiffness of a structurally damaged system.

### 6.1. Kalman Filtering Approach

As will be seen in the numerical example simulation given in Section 7, the usefulness of the OTSKE (or the ASKF) can be compromised if the chosen *Q*_*k*_^*d*^ is unproper, by assuming an improper unknown input model, and thus the damage stiffness may not be correctly identified. Specifically, the estimated damage stiffness of the OTSKE may not work well for the health stiffness; that is, *δk*_*i*_ = 0, by choosing a large value of *Q*_*k*_^*d*^. On the other hand, the damage stiffness may also not be correctly estimated if a small value of *Q*_*k*_^*d*^ is chosen. To address this problem, a multiple model approach similar to that given in [26] may be used. Thus, in this subsection we present a structural damage detection method based on the multiple model approach via the OTSKE to improve the identified damage stiffness results.

First, we use a small value of *Q*_*k*_^*d*^, denoted as *Q*_*k*_^*d*^ = *γ*_*s*_ *I*_*n*_, in the OTSKE to detect (identify) those places that the stiffness are in health. Let *ϵ*(*γ*_*s*_) be a given dead-zone threshold associated with the small value *γ*_*s*_. Then, the *i*th area with healthy stiffness is declared when the *i*th output of the unknown input estimator ${\hat{d}}_{k}^{i}$ satisfies the following relationship: $\left|{\hat{d}}_{k}^{i}|\leq \epsilon (\gamma_{s}\right)$, and hence the corresponding damage stiffness detection value is set to zero. On the other hand, if the *i*th output of the unknown input estimator ${\hat{d}}_{k}^{i}$ satisfies the relationship: $\left|{\hat{d}}_{k}^{i}|>\epsilon (\gamma_{s}\right)$, then we set the corresponding damage stiffness detection value to one.

Second, we use a large value of *Q*_*k*_^*d*^, denoted as *Q*_*k*_^*d*^ = *γ*_*l*_ *I*_*n*_, where *γ*_*l*_ > *γ*_*s*_, in the OTSKE to estimate the true damage stiffness, denoted as ${\hat{d}}_{lk}$.

Finally, we denote a detection matrix *D*_*k*_ as

(60) $$\begin{array}{c} D_{k}={\left[\begin{array}{cccc} D_{k}^{1} & D_{k}^{2} & \cdots & D_{k}^{n} \end{array}\right]}^{T}, \end{array}$$

where *D*_*k*_^*i*^ is given as follows:

(61) $$\begin{array}{c} D_{k}^{i}=\{\begin{array}{cc} 0 & \left|{\hat{d}}_{k}^{i}\right|\leq \epsilon \left(\gamma_{s}\right),\\ 1 & \left|{\hat{d}}_{k}^{i}\right|>\epsilon \left(\gamma_{s}\right), \end{array} \end{array}$$

and the estimate of the *i*th damage stiffness is obtained as follows:

(62) $$\begin{array}{c} {\hat{d}}_{k}^{i}=\min\left(D_{k}^{i},{\hat{d}}_{lk}^{i}\right). \end{array}$$

### 6.2. Constrained Optimization Approach

First, we address the damage detection issue. Using (10), (50), (55), (56), and (59), we obtain the following alternative damage stiffness estimate:

(63) $$\begin{array}{c} {\hat{d}}_{k}=\psi_{k}^{+}S_{k}^{*}\left({\hat{H}}_{k}d_{k}+\Phi {\tilde{X}}_{k\;|\;k-1}+v_{k}\right), \end{array}$$

where

(64) $$\begin{array}{c} S_{k}^{*}={\left(S_{k}^{T}{\tilde{R}}_{k}^{-1}S_{k}\right)}^{+}S_{k}^{T}{\tilde{R}}_{k}^{-1},\\ {\tilde{X}}_{k\;|\;k-1}=X_{k}-{\hat{X}}_{k\;|\;k-1}. \end{array}$$

As shown in [22], under the following condition:

(65) $$\begin{array}{c} \mathrm{rank}\left[S_{k}\right]=\mathrm{rank}\left[{\hat{H}}_{k}\right]+\mathrm{rank}\left[{\overset{-}{\hat{U}}}_{k-1}\right], \end{array}$$

the expectation of (63) is given as follows:

(66) E[d^k]=(ψkTψk)−1ψkTSk∗(H^kE[dk]+ΦU^−k−1E[d~k−1]),=(ψkTψk)−1ψkTSk∗Sk[φkE[dk]U^~k−1E[dk−1]]≈(ψkTψk)−1ψkTψkE[dk]=E[dk].

It is thus clear from (66) that the *i*th component of the signal ${\hat{d}}_{k}$ will be zero only if the *i*th stiffness is healthy, which also signifies that the *i*th element of the damage stiffness estimate will behave like a zero-mean white noise.

Next, we address the damage identification issue. Because the constrained optimization approach is more sensitive than the Kalman filtering approach, and those components of ${\hat{d}}_{k}$ that are healthy are zero-mean white noises, the extent of the damage stiffness can be identified using the time average of the damage stiffness estimates as follows:

(67) $$\begin{array}{c} \delta k_{i}=E\left[{\hat{d}}_{k}^{i}\right]\approx \frac{1}{k}\sum_{j=1}^{k}{\hat{d}}_{j}^{i}. \end{array}$$

Finally, in order to decrease the noise effect and to increase the robustness of the above identification algorithm, we modify (67) by incorporating a suitable chosen threshold *ϵ* as the following effective mean:

(68) $$\begin{array}{c} \delta k_{i}=E{\left[{\hat{d}}_{k}^{i}\right]}_{|{\hat{d}}_{k}^{i}|\leq \epsilon }. \end{array}$$

## 7. A Numerical Example

### 7.1. 6-Storey Shear Building Model

In this paper, an example study for the detection of the location and the identification of the extent of damage stiffness matrix of a six-storey shear building is provided to illustrate the effectiveness of the proposed results. The shear building is a simplified model that assumes all of the building mass is lumped at the floor. The floor and beams of the shear building are rigid relative to its column. Therefore, there are only lateral displacements and no axial deformation or rotation. The displacements at each floor are defined by one degree of freedom alone. Thus, there are six degrees of freedom in the structure to describe total displacements in the considered example.

The parameters of system (1) in this study are chosen as *m*_1_ = *m*_2_ = ⋯ = *m*_6_ = 30 Kg and *k*_1_ = *k*_2_ = ⋯ = *k*_6_ = 55500 N/m. The chosen damping matrix is Rayleigh damping, based on the assumption that the damping ratio is chosen as 5% for all modes. The damage is assumed to occur at the first three storeys, and all instances of damage are modeled as a 50% reduction in stiffness. In the simulation, we assume that only acceleration sensors are used. Thus, using *H*_*d*_ = *H*_*v*_ = 0 and *H*_*a*_ = *I* in (6) we have the following acceleration response:

(69) $$\begin{array}{c} y=\ddot{x}=-M^{-1}C\dot{x}-M^{-1}Kx-L{\ddot{x}}_{g}-M^{-1}F\left(x\right)d, \end{array}$$

which yields the following measurement matrices:

(70) $$\begin{array}{c} \Phi =-M^{-1}\left[\begin{array}{cc} K & C \end{array}\right],\;\;D=-L,\\ H=-M^{-1}F\left(x\right). \end{array}$$

**Figure 1:**
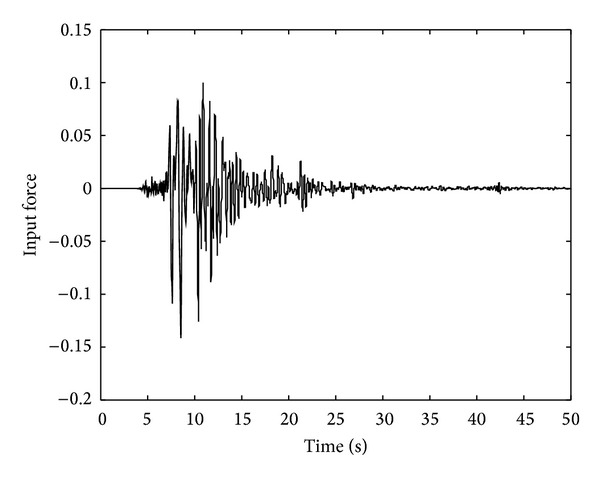
Ground acceleration excitation.

**Figure 2:**
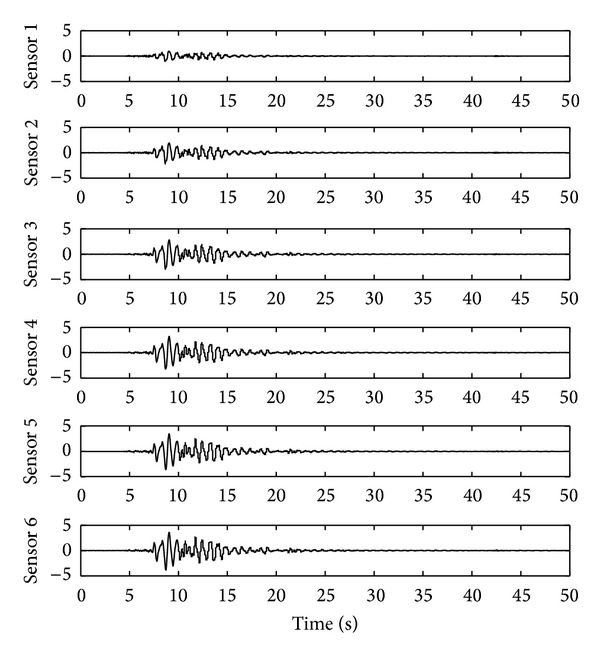
Acceleration sensor responses.

The sampling rate for the measurement is chosen as 100 Hz. The ground acceleration excitation is illustrated in Figure 1, and the response of the acceleration sensor in each floor is shown in Figure 2. In this simulation, both the OTSKE and the RTSKF are considered. The initial setting of the OTSKE is given as follows: ${\overset{-}{X}}_{0|-1}=0$, *d*_−1∣−1_ = 0, *U*_0_ = 0, *Q*_0_^*X*^ = diag⁡{0, *I*}, $P_{0|-1}^{\overset{-}{X}}=I$, and *P*_−1∣−1_^*d*^ = 0, while that for the RTSKF is given as follows: ${\hat{X}}_{0|-1}=0$ and *P*_0∣−1_^*X*^ = *I*. The measurement noise covariance is set as *R*_*k*_ = *I*.

### 7.2. Damage Detection and Identification Using the OTSKE

**Figure 3:**
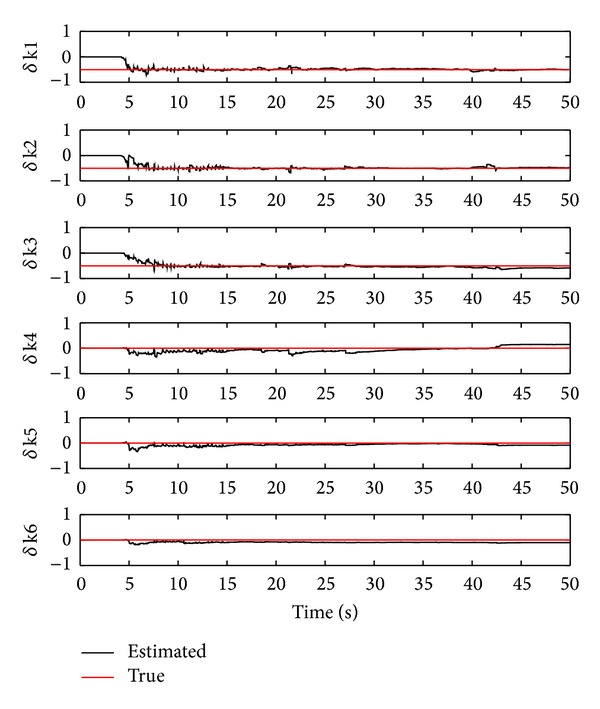
Damage stiffness estimation using the OTSKE with *Q*_*k*_^*d*^ = 1000*I*_6_.

The variation of the stiffness of each storey can be identified using Kalman filtering with a suitable chosen *Q*_*k*_^*d*^.  Figure 3 shows the identified damage stiffness values and the true ones using the OTSKE with *Q*_*k*_^*d*^ = 1000*I*_6_. From the figure, it is clear that the damage stiffness of the first three storeys can be correctly identified with the following error percentages: 0.41%, 3.83%, and −16.88%, respectively. On the other hand, the estimated damage stiffness values of the 4th to 6th storeys differ from their true values, yielding −0.15, 0.09, and 0.11 reductions of their corresponding true stiffness values. This simulation clearly illustrates that the OTSKE can accurately estimate the damage stiffness. However, it may also fail to identify healthy stiffness values.

**Figure 4:**
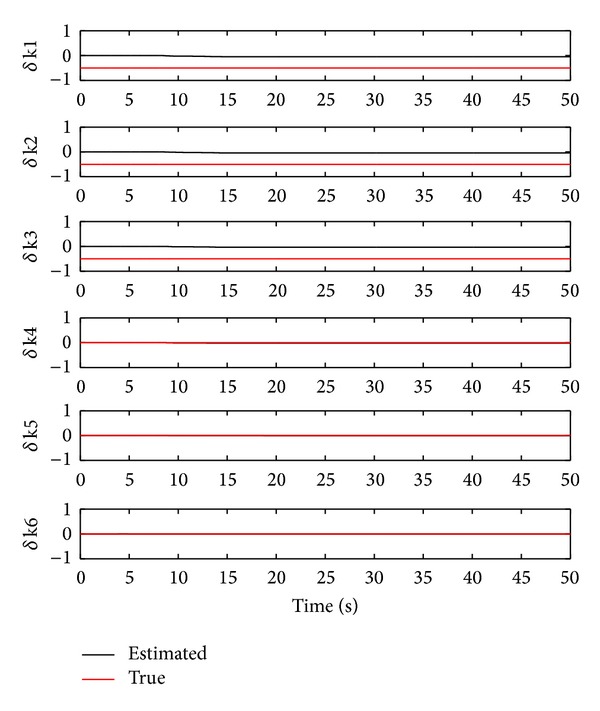
Damage stiffness estimation using the OTSKE with *Q*_*k*_^*d*^ = 0.001*I*_6_.

In showing one possibility of correctly identifying healthy stiffness values using the OTSKE, we decrease the covariance *Q*_*k*_^*d*^ as *Q*_*k*_^*d*^ = 0.001*I*_6_. The identified damage stiffness values are illustrated in Figure 4, from which the estimated damage stiffness values of the 4th to 6th storeys are near their true values, yielding 0.02, 0.01, and 0.00 reductions of their corresponding true stiffness values. However, it is also observed that in this case the damage stiffness values of the first three storeys are identified with the following respective error percentages: 90.08%, 90.81%, and 93.02%, which obviously differ from their true values. This simulation case clearly shows the potential disadvantage of applying the OTSKE to structural damage estimation.

**Figure 5:**
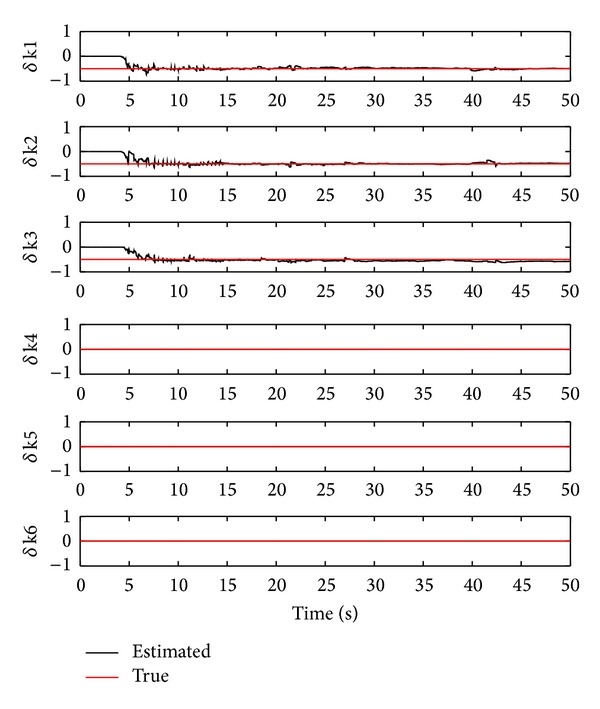
Damage stiffness estimation using the OTSKE with *Q*_*k*_^*d*^ = diag⁡{1000*I*_3_, 0.001*I*_3_}.

From the above simulation results, we may draw the following implications: (1) the larger the value of the covariance matrix *Q*_*k*_^*d*^ chosen is, the more accurately the extent of the damage stiffness will be identified, and (2) the smaller the value of the covariance matrix *Q*_*k*_^*d*^ chosen is, the more accurately healthy stiffness areas will be detected. With the above observations, if one can correctly identify the places which have damage stiffness, that is, the first three storeys, and identify healthy areas, that is, the 4th to 6th storeys, then one can properly choose *Q*_*k*_^*d*^ as *Q*_*k*_^*d*^ = diag⁡{1000*I*_3_, 0.001*I*_3_}.  Figure 5 shows the identified damage stiffness values and the true values using this specific *Q*_*k*_^*d*^, from which we observe that the estimated damage stiffness values of the 4th to 6th storeys are very near their true values, with 0.00, 0.00, and 0.00 reductions of their corresponding true stiffness values. Moreover, the damage stiffness values of the first three storeys are identified with the following respective error percentages: 0.13%, 4.05%, and −15.00%. Nevertheless, it should be stressed that this specific value of *Q*_*k*_^*d*^ is heuristically chosen and may not work well in practical system design.

**Figure 6:**
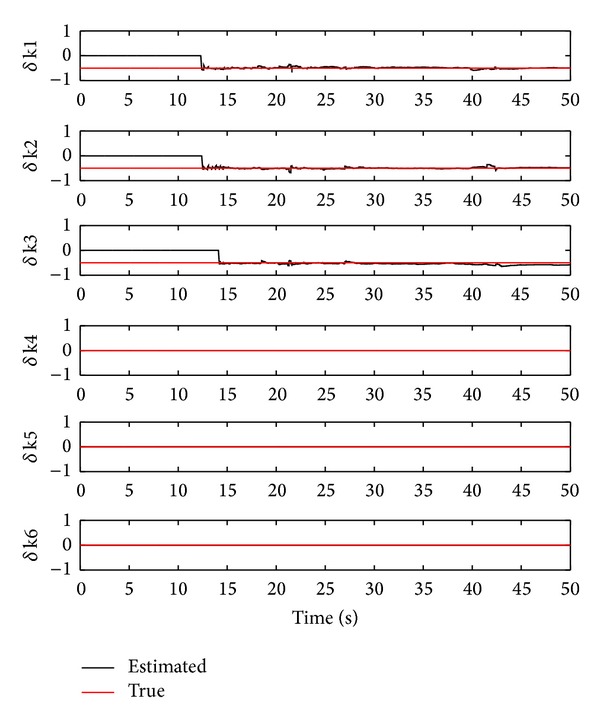
Damage stiffness estimation using multiple model approach (*γ*_*s*_ = 0.001, *γ*_*l*_ = 1000, and *ϵ*(*γ*_*s*_) = 0.03).

In order to improve the aforementioned potential shortcoming of the OTSKE, in the following we show an alternative application of the new proposed multiple model approach via the OTSKE in Section 6.1 in order to identify the damage stiffness of all storeys. To achieve this aim, we choose *γ*_*s*_ = 0.001, *γ*_*l*_ = 1000, and *ϵ*(*γ*_*s*_) = 0.03.  Figure 6 shows the identified stiffness and the true values using the multiple model approach, from which we obtain that the estimated damage stiffness values of the 4th to 6th storeys correspond to exactly their true values, which are zero. Moreover, the damage stiffness values of the first three storeys are identified with the following respective error percentages: 0.41%, 3.83%, and −16.88%, which are slightly poorer than those obtained using the OTSKE with *Q*_*k*_^*d*^ = diag⁡{1000*I*_3_, 0.001*I*_3_}. However, it should be stressed that no information on the damaged storeys is required for the proposed multiple model approach. Note that the feasible value of the dead-zone threshold *ϵ*(*γ*_*s*_) remains to be determined.

### 7.3. Damage Detection and Identification Using the RTSKF

As shown in the previous subsection, the location and the extent of damage stiffness may not be simultaneously identified by a single filter when applying the conventional Kalman filtering approach, for example, the OTSKE. In this subsection, we demonstrate the application of the proposed RTSKF to detect the locations in which the stiffness is healthy and to identify the extent of any damage stiffness.

**Figure 7:**
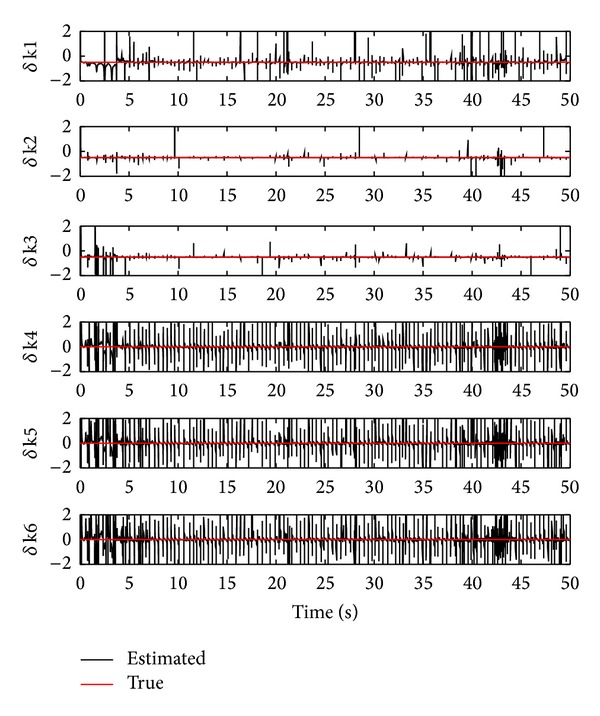
Unknown inputs estimation using the RTSKF.

First, we show the detection simulation results using the RTSKF. To this end, we illustrate the unknown input estimates, that is, ${\hat{d}}_{k}$, in Figure 7. From the figure, we find that the magnitudes of the stiffness estimates of the last three storeys, which are healthy, are more like white noise than those of the others. Therefore, we deduce that the storey which has no damage stiffness may yield a white-noise-like signal. Based on the above observed results, one can correctly detect the storey with no damage stiffness. In this simulation, a numerical measure is further used to quantitatively determine the location of damage stiffness. The quantitative measure is defined as a cumulative excess of magnitude bound, represented by *E*_MB_. For the *i*th damage stiffness, *E*_MB_^*i*^ is given as follows:

(71) $$\begin{array}{c} E_{\text{MB}}^{i}=\sum_{k}u_{s}\left(\left|{\hat{d}}_{k}\left(i\right)\right|-\epsilon \right), \end{array}$$

where *u*_*s*_ is the unit-step function. By choosing *ϵ* = 1.5, the values of *E*_MB_ for 1 to 6 storeys are given by 78, 13, 20, 211, 221, and 237, respectively, which gives a more clear indication that the last three storeys have no damage stiffness.

**Figure 8:**
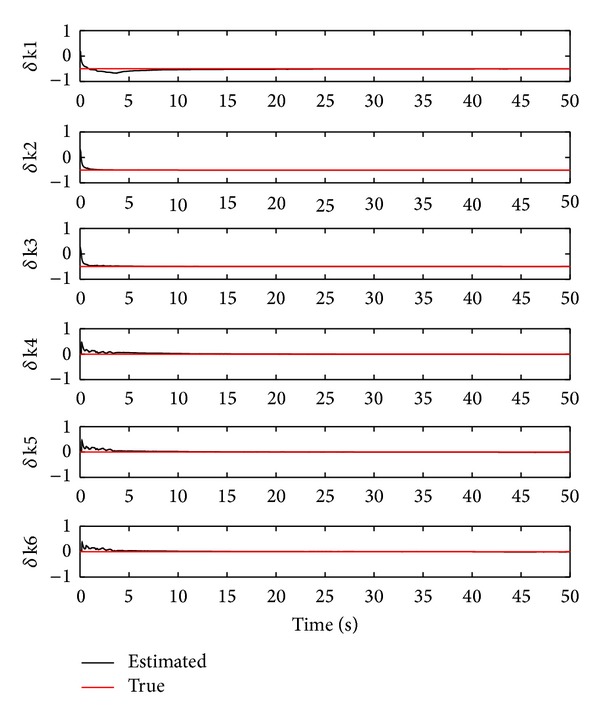
Damage stiffness estimation using the RTSKF (*ϵ* = 1.5).

Second, we show the identified extent of the damage stiffness based on the effective mean algorithm (68). This is illustrated in Figure 8. From the figure, we obtain that the estimated damage stiffness values of the first three storeys are given by −0.5045, −0.5062, and −0.5035, respectively, which have the following respective error percentages: −0.91%, −1.24%, and −0.70%. These identified results are slightly better than those obtained using the multiple model approach via the OTSKE. Furthermore, the estimated damage stiffness values of the last three storeys are given by −0.0082, −0.0121, and −0.0104, respectively. These identified results are comparable to those obtained using the multiple model approach via the OTSKE.

In summary, the above simulation results illustrate the usefulness of the proposed damage detection and identification algorithm through the proposed constrained optimization approach.

### 7.4. Discussions

As shown in the previous two subsections, the Kalman filtering approach serves as a useful means of simultaneously detecting the healthy areas and identifying the extent of any damage stiffness. It should be stressed that, as compared to the substructure-based FRF approach in [6], this time-domain-based method is a direct approach to solving damage detection and identification problems in the sense that no further frequency domain transformations are used. Moreover, apart from the approach in [6], where only the locations of damage stiffness can be detected, the proposed optimal filtering algorithms can accurately estimate the extent of any damage stiffness.

**Figure 9:**
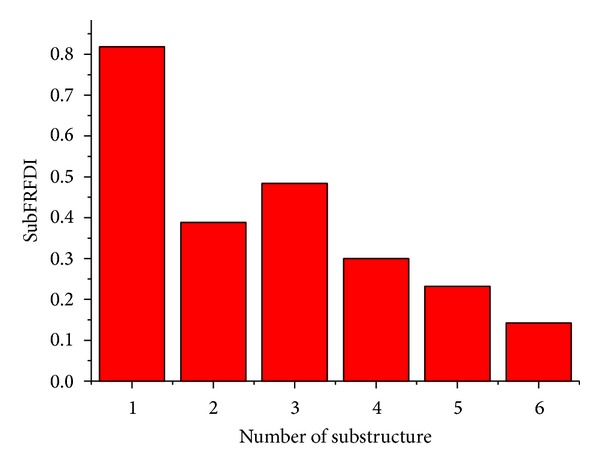
Damage location indices obtained through a substructure-based FRF approach.

In order to compare the detection performance of the proposed RTSKF with that of the method in [6], we further consider the substructure-based FRF approach with a damage location index (SubFRFDI) in order to locate damage. In this method, a multi-DOF (degrees of freedom) structure is divided into several substructures. Thus, for the simulation case, we have six substructures. Then, the FRF of each substructure is calculated in order to obtain the dedicated SubFRFDI. If the properties of a structural system do not change, then the index is close to zero. However, if the damage to a specific storey of the shear building is severe, then its corresponding index value will be high. We illustrate the SubFRFDI values of the considered six-storey shear building model in Figure 9. From the figure, it is obtained that the SubFRFDI values of the last three storeys are smaller than those of the first three storeys, which indicates that the damage to the first three storeys is more severe than that of the others. However, it is also observed that the SubFRFDI values of the 4th and 5th storeys may be high enough to be declared to have damage stiffness. Note that the SubFRFDI-based method lacks the ability to determine the damage level. On the other hand, the proposed optimal filtering methods can simultaneously identify the extent of the damage stiffness and detect those places where the stiffness is healthy, which shows that they are more reliable than those obtained through the substructure-based FRF approach.

## 8. Conclusion

This paper has presented a novel state-space-based structural damage estimation technique, based on discrete-time Kalman filtering and unbiased minimum-variance filtering (or constrained optimization method), to detect areas where the stiffness is healthy and to identify the extent of any damage stiffness. It is shown by a numerical example that the previously proposed OTSKE with a multiple model approach can accurately estimate the damage stiffness and detect healthy stiffness. Moreover, this paper shows that the recently proposed EDKF can also be applied to detecting and identifying structural damage. A special implementation of the EDKF, called the RTSKF, is also proposed in order to reduce computational complexity. Through the RTSKF, the new proposed quantitative measure *E*_MB_, and the effective means of the damage stiffness estimates given by (68), one can more accurately detect and identify damage stiffness. Simulation results show that the proposed optimal filtering methods are more reliable than those obtained through the substructure-based FRF approach in the sense that both the location and extent of damage stiffness are provided in the former.
